# A FEM-Based 2D Model for Simulation and Qualitative Assessment of Shot-Peening Processes

**DOI:** 10.3390/ma14112784

**Published:** 2021-05-24

**Authors:** Georgios Maliaris, Christos Gakias, Michail Malikoutsakis, Georgios Savaidis

**Affiliations:** 1 Department of Chemistry, School of Sciences, International Hellenic University, 65404 Kavala, Greece; 2 Laboratory of Machine Elements & Machine Design, School of Mechanical Engineering, Aristotle University of Thessaloniki, 54124 Thessaloniki, Greece; clgakias@auth.gr (C.G.); mmalikou@auth.gr (M.M.); gsavaidis@auth.gr (G.S.)

**Keywords:** shot peening, finite element method, modeling, simulation, shot dynamics, residual stresses, surface roughness

## Abstract

Shot peening is one of the most favored surface treatment processes mostly applied on large-scale engineering components to enhance their fatigue performance. Due to the stochastic nature and the mutual interactions of process parameters and the partially contradictory effects caused on the component’s surface (increase in residual stress, work-hardening, and increase in roughness), there is demand for capable and user-friendly simulation models to support the responsible engineers in developing optimal shot-peening processes. The present paper contains a user-friendly Finite Element Method-based 2D model covering all major process parameters. Its novelty and scientific breakthrough lie in its capability to consider various size distributions and elastoplastic material properties of the shots. Therewith, the model is capable to provide insight into the influence of every individual process parameter and their interactions. Despite certain restrictions arising from its 2D nature, the model can be accurately applied for qualitative or comparative studies and processes’ assessments to select the most promising one(s) for the further experimental investigations. The model is applied to a high-strength steel grade used for automotive leaf springs considering real shot size distributions. The results reveal that the increase in shot velocity and the impact angle increase the extent of the residual stresses but also the surface roughness. The usage of elastoplastic material properties for the shots has been proved crucial to obtain physically reasonable results regarding the component’s behavior.

## 1. Introduction

Shot-Peening (SP) is a widely applied surface treatment processes to improve the fatigue performance of engineering components made of metallic materials. Thereby, many tiny particles (shots) bombard the surface of the component with high velocity. The impacts deform plastically the component’s surface leading to a pronounced compressive residual stress state at the surface layers which act beneficially to its fatigue performance, since they increase the surface resistance to crack initiation, reduce the crack propagation rate, and may even lead to short crack arrest [1,2]. In addition, the repeated impacts lead to work hardening of the surface layers and increase the local flow stress and, therewith, the resistance to fatigue loads. On the other hand, the SP process inevitably affects the surface roughness and, therewith, the fatigue life on a negative way. Valuable overviews on the individual effects and their mutual interactions are given in [3,4,5,6], among other sources. 

The development of industrial SP processes for new material–component combinations or its optimization for existing ones is a complicated task, since many variables are involved, with the most important being shot velocity, shot size(s), shot size distribution, shot material properties, impact angle, and coverage. The related industry is based on years-long experience and long-lasting experimental works to accomplish this task. The demand for capable, targeted, and user-friendly theoretical SP simulation models to support this task and reduce the experimental effort is growing. The continuous increase in computational capacity and appearance of capable and accurate commercial software over the past decades conduced to come closer to the industrial demands. Remarkable progress has been achieved, starting from the simulation of single shots on bodies and the formation of corresponding contact stresses as provided, e.g., in [7,8], the work of Zion and Johnston [9] introducing a 2D Finite Element Method-based simulation model capable to consider various shot types and Al-Hassani et al. [10] considering multiple shots. Extensive investigations followed to further study and model the physical phenomena of multiple shot impacts at surfaces using either 2D or 3D models [3,4,11,12]. In the last decade, FEM-based simulation models have been proposed, aiming to describe the fundamental processes taking place during a shot-peening treatment and explain and predict the correlation between the SP parameters and the process results. Among others, valuable assistance regarding the numerical modeling and simulation of SP processes in reference to the residual stress entrapment, the resulting surface roughness, and cold-work extent are contained in [13,14]. 

Despite the progress achieved, decisive issues regarding the elastoplastic shot material behavior, shot size distribution(s), and coverage aspects are still not sufficiently covered. The present paper settles at this point aiming to bridge these gaps. A Finite Element-based 2D SP model has been developed within the framework of the LIGHTTECH-project [15], capable of covering the aforementioned issues with sufficient accuracy. Having a solid elastoplastic material mechanics background, the model aims at providing precious insight into the effects and interactions of the SP parameters on the surface properties (residual stress state and roughness) of the part under treatment. Due to its 2D nature, it allows quick execution of process simulations, accurate comparison, evaluation, and selection of promising process parameters, before proceeding to computationally extensive and time-consuming 3D simulations and/or long-lasting and costly experimental trials. The essential novelty of the developed model is its capability to consider real size distributions and elastoplastic material properties for the shots—issues that constitute major lacks in current models. Therewith, the new model aims at physically more reasonable and more accurate results regarding the effects of individual SP parameters, approaching the ultimate target of the industry to reduce developing times and costs of SP processes and increase their efficiency. However, one should consider the restrictions and limitations always entailed in a 2D-mode, which affect the calculated quantities of the residual stresses and the roughness. This is because actual point contacts between component surface and shot at impact are unavoidably modeled through line contacts in a 2D model. 

Furthermore, the model has been utilized to study the influence of serial shots and their size distribution, their impact velocity, and angle on the surface properties of leaf springs used for the suspension of heavy truck axles. For this, S460 steel shots and shot size distributions, as used in serial SP processes of top European leaf spring manufacturers that participated in [6], have been analyzed and considered within the framework of this paper. The qualitatively accurate simulation results confirm the effectiveness of the developed 2D model.

## 2. Finite Element Analysis Model

The developed model employs some unique characteristics such as variable shots’ diameter, which are modeled applying a stochastic approach, as well as the definition of nonlinear material properties for the shots. The following subsections provide insights into the model’s structure and elements:

### 2.1. Impact Shots Geometry Generation

**Figure 1:**
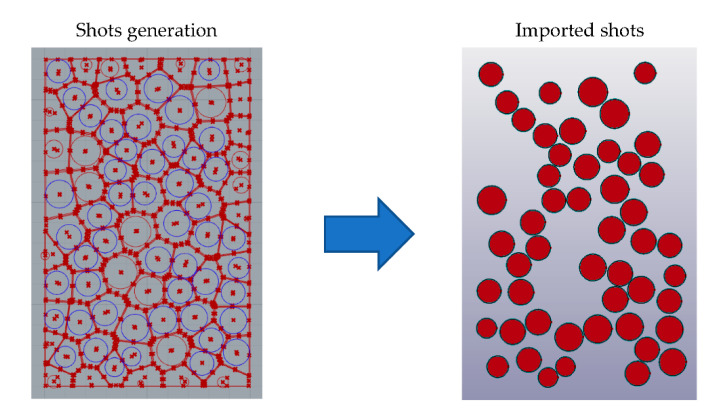
Shot generation using the Voronoi-based algorithm (on the left) and imported shots (shown as blue circles on the left) in the pre-/post-processor (on the right).

Shots used in industrial SP processes exhibit slightly varying diameters, with values lying in between a specific range, which depends on their type. Since every shot has a different diameter, it is necessary for the model to be capable of matching the size distribution of the S460 steel shots used in an industrial application. The abovementioned requirement led to the development of a python algorithm, which is based on the Voronoi tessellation method [16]. The specific process is illustrated in Figure 1. 

A specific area above the specimen is divided into random polygons, and then the geometric center of every polygon is used for drawing a circle which represents a single shot. The diameter of each circle is calculated as a percentage of the minimum vertical distance between the center of the corresponding polyhedron and its edges. By altering the initial seed points number (which determine the number of the generated polygons), it is possible to statistically match the diameter distribution of the real shots. The nominal size of the specific shots used here for the parametrical studies (type S460) was 1.4 mm. Furthermore, the specific method orients the shots in a random way on the 2D plane. The shots which conform to the defined parameters are then exported to the FEA software.

The real S460 shots studied in the present investigation have a spherical shape. The diameter distribution of the real shots is determined by sieve analysis according to SAE J444 standard [17]. Relative information is also provided by metal blasting abrasives manufacturers, e.g., Kholee Blast [18] and Metal White [19]. The mass of shots passing through each sieve level is measured, and the percentage mass of the total is calculated. Statistical analysis is performed correlating the data from the standard sieve analysis with the diameter range of each sieve level. This results in a representative standard deviation of the shot diameter of 0.135 mm, while considering the nominal size of 1.4 mm as a mean diameter of the specific shot type. In addition, there is no important indication that the diameter follows a Gaussian (normal) distribution since the sieve levels are not equally spaced, but for reasons of empirical knowledge, the Gaussian approximation was adopted for the FE modeled shots. Table 1 shows the comparison between the standard sieve analysis results and the corresponding classification data for the generated shots.

### 2.2. FEA Mesh and Constraints 

The geometry of the FEA model represents a portion of the real part, here with dimensions of 20 mm of length by 5 mm of height. Both dimensions have been carefully chosen considering the requirement that the developed stress field must not reach the model’s boundary. After geometry definition, the next step is the discretization of the model using finite elements. This is a crucial step since meshing parameters such as element size, total element number, and element quality affect the processing effort, the total simulation time, the accuracy, and the quality of the obtained results. To find a common denominator between element size and solution time, a variable element size scheme has been employed. From prestudies carried out, two important conclusions were drawn regarding the element length. The first was that the shots and the surface layers must have the same element length to improve the accuracy of the contact calculation algorithm. The second concerns the convergence of results for various element lengths. It turned out that a discretization finer than an element length of 20 μm slightly improves the accuracy but decreases the required computational time significantly.

**Figure 2:**
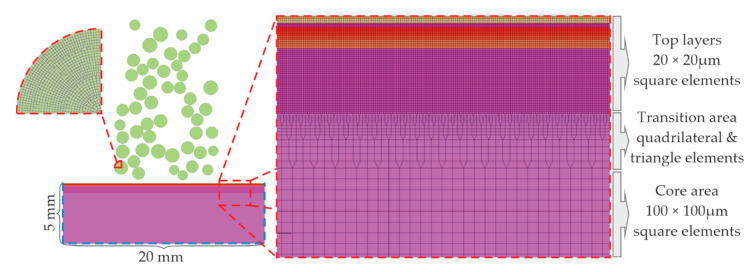
Specimen discretization strategy.

Objects or areas where large deformations are expected must be discretized with smaller elements to improve the accuracy of the FE-assisted calculations and, consequently, the extracted residual stresses profiles. On the other hand, model parts, which are away from the impact surface, can be meshed with larger elements to reduce the computational time. The final dimensions of the generated elements are determined after the conduction of element convergence studies. The dominant element shape is square, and the element aspect ratio should be as close as possible to 1 to avoid convergence issues during solution. The result of meshing strategy is depicted in Figure 2.

As shown in Figure 2, the specimen is divided in three areas with respect to the specimen’s height: the upper (“Top layers”) area, the bottom (“Core”) area, and another one in between (“Transition” area). The “Top layers” area is discretized with square elements having an edge length of 20 μm, the “Core” area again with square elements having an edge length of 100 μm, and the “Transition” area with quadrilateral and triangle-shaped elements with dimensions varying between 10 and 100 μm. The applied elements are two-dimensional shell elements with plane strain formulation for specimen and shots. The shots were also discretized using an element size of 20 μm. The mesh of a quarter of a shot is illustrated on the left-hand side of Figure 2. 

Regarding the boundary and initial conditions, the nodes of the specimen’s vertical edges are restrained in the horizontal (X) direction, where the nodes of the specimen’s bottom edge are restrained in the vertical (Y) direction, as it is pictured on the bottom left-hand side in Figure 2 with cyan dashed lines. Notice that one issue that arose during the initial simulations was the appearance of artificial stress waves generated during the impacts of the shots on the specimen’s surface. These waves were traveling inside the specimen, bouncing on its boundaries, and, thus, contaminating the results. The solution given was the definition of a damping boundary condition, which is applied on the nodes of the boundary of the specimen and permits the absorption of the artificial stress waves in between few simulation steps. In- depth information for the specific boundary condition is given in the next section. The initial velocity of the shots ranged from 35 to 77 m/s, at an impact angle between 45 and 90° with respect to the horizontal (X) axis. A contact scheme was selected for the contact during the impacts between the shots and the specimen. Friction as well as interactions between the shots were neglected. Notice that in the case of friction, a simulation was conducted where a friction value of 0.2 between the shots and the specimen was set. The assessment of the results revealed that the effect of friction on the calculated parameters is negligible, but the impact on solution time is countable. Due to the involved dynamic effects of the SP process (linear velocity of the shots is larger than 10 m/s), the use of an explicit FEA solver is mandatory. In Table 2, the main FE model inputs are summarized.

### 2.3. Stress Wave Damping

Since high velocity impact and specifically shot peening is a strictly dynamic phenomenon, random oscillations and stress waves during and after the impact need to be controlled. To prevent these phenomena, artificial material damping has been considered for the specimen using Rayleigh damping type, which is a function of mass and stiffness characteristics of the modeled material. Specifically, it is described by the Equation (1):

C = α·M + β·K,(1)

where C is the damping matrix, M is the mass matrix, α represents a mass proportional coefficient, K stands for the stiffness matrix, and β is a stiffness proportional coefficient. 

The correlation between fraction of critical damping, ζ_i_, and the damping factors α and β is:

ζ_i_ = α/2ω_i_ + β ω_i_/2, (2)

with ω_i_ being the natural frequency for a specific mode. According to Ghasemi [20] and Mequid [21], the value ζ = 0.5 has been selected for rapid damping of unwanted oscillations and to avoid any computational instability. Since the minimal modal frequency, ω_0_, is governing the oscillating behavior of the specimen after the impact, the damping parameters a and β should be selected, taking that mode into account, which can be estimated as:

ω_0_ = 1/h·√(2E/ρ), (3)

where E and ρ are the Young’s modulus and the density of the specimen material, respectively, and h is the height of the specimen. 

After setting β = 2.1 × 10^−6^ ms, the value α = 2071.5 rad/ms has been calculated. Although, a sufficient number of trial runs was required to validate and fine-tune both of the parameters.

### 2.4. Material Properties Definition

**Figure 3:**
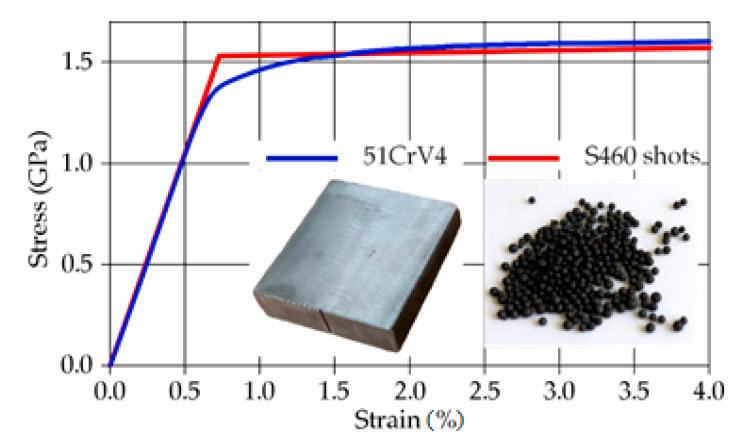
Stress–strain curves for the specimen (blue colored) and the shots (red colored).

The stress–strain behavior of the spring steel under consideration here, 51CrV4, has been determined through tensile tests, and is presented in Figure 3 with the blue colored curve. The yield stress (stress at 0.2% of plastic strain) and ultimate tensile strength amount to 1450 and 1645 MPa, respectively. 

Considering information from steel shot manufacturers, S460 shots follow the SAE J827 [22] and ISO 11124/3 [23] specifications regarding the chemical composition, hardness, microstructure, and physical characteristic requirements. Their stress–strain behavior can be accurately approximated by a bilinear curve [24]. The bilinear approximation used in the present study is illustrated in Figure 3 with red color. The yield stress has been estimated through nanoindentation experiments [25] 1500 MPa. The tangent modulus has been derived from the difference between the yield stress and the ultimate tensile strength (1700 MPa) of the shots. Notice that the latter value has been converted from the maximum Rockwell-C value (here given to 51 HRC from the shot manufacturer for the specific shot type) following the DIN 50, 150 standard [26]. Table 3 and Table 4 summarize the key mechanical properties and the chemical composition of the part and shot materials. 

Regarding the material properties of the shots, the present study implements the elastoplastic stress–strain of the steel shots, as shown in Figure 3. However, this is not a common act in the literature. Most researchers use totally rigid (totally non-deformable) shots in FE shot-peening simulations. It should be mentioned that modeling steel shots with rigid material characteristics is sufficient and leads to accurate results for SP simulations with large stiffness difference between the two interacting materials, e.g., at SP of aluminum alloyed parts. However, in the present work, the steel shots and the peened part have similar properties. Therefore, a rigid shot approach may lead to significant errors. 

Both materials follow a purely isotropic hardening rule, according to the model of Krieg and Key [27] for time-dependent plasticity theory. The slope of the stress–strain curve during the unloading procedure, when reverse yielding occurs, is defined by the plastic hardening modulus H, which is a function of Young’s Modulus (E) and tangent modulus (E_t_), specifically:

H = E × E_t_/(E − E_t_). (4)

The impact velocity, strain rate, and viscosity effects have been explicitly considered by means of the Cowper–Symonds material model [28]. The yield stress is scaled by the factor

(5) $$q=1+(\dot{\varepsilon }\;\times C{)}^{\left(1/P\right)}$$

where $\dot{\varepsilon }$ is the strain rate, and C, P are the Cowper–Symonds parameters. They have been experimentally determined in [29] for an equivalent 51CrV4 lot and adapted to the lot of the current study. Hence, the values of Cowper–Symonds parameters used here amount to C = 1.1 × 10^13^ ms^−1^ and P = 9.6. Notice that high strain rate values up to 100 ms^−1^ were obtained in the present study.

## 3. Parametrical Studies 

The developed 2D model has been applied to study the effect of two important parameters, the shot velocity and the impact angle, on the residual stress and roughness profiles. In the context of the evaluation of the simulation, a systematic way was required to extract a representative residual stress profile for the peened area, since local variations of the stress results are always apparent due to the stochastic nature of the SP simulation. A python script was developed, which averages the stress values and other field variables of the peened area, up to a depth of 0.9 mm (=height of the top layers). The standard deviation and the average stress are used as an indication of the uniformity of the residual stress field.

The characteristic roughness values are extracted from the displacement of the surface nodes of the peened area in X and Y directions. These displacements can be easily visualized, and characteristic roughness parameters according to DIN EN ISO 4287 [30] can be calculated. The evaluation length amounted to L = 10 mm (±5 mm from the center of the part shown in Figure 2). The selected roughness parameters are Total Height R_t_, Mean Roughness R_a_, Root Mean Square of roughness R_q_, and the Average Depth of roughness R_z_. 

R_t_ represents the vertical distance between the highest peak and the deepest valley within the evaluation length. R_a_ and R_q_ are calculated according to Equations (6) and (7), respectively.

(6) $$R_{a}=(1/L)\;\times \;\int_{0}^{L}\left|Z\left(x\right)\right|\;\mathrm{dx}$$

(7) $$R_{q}=[(1/L)\;\times \;\int_{0}^{L}Z{\left(x\right)}^{2}\;\mathrm{dx}{]}^{1/2},$$

where by Z(x) stands for the profile height function. For the calculation of R_z_, the evaluation length L has been divided into five equal sections, i = 1–5, each of 2 mm length, and the Total Height, R_t,i_, of each section has been determined. R_z_ stands for the arithmetic average value of these five Total Heights according to Equation (8)

(8) $$R_{Z}=1/5\cdot \Sigma R_{t,i}\;i=1\mathrm{to}5.$$

### 3.1. Impact Velocity

**Figure 4:**
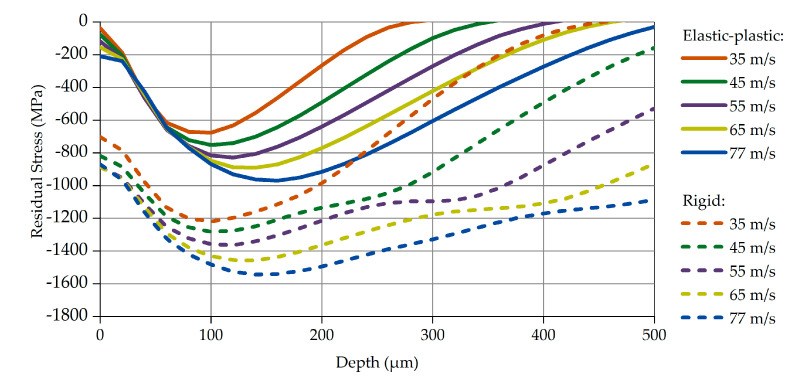
Compressive Residual Stress profiles, for various impact velocities.

To capture the effect of the shot velocity, the shots were simulated to impact the part’s surface vertically. Different velocities were tested, in a range from 35 to 77 m/s, which is of specific interest to the automotive leaf spring industry. Since the exact condition of the shots in the serial SP application varies and cannot be precisely defined, both shot conditions, elastic-plastic and rigid, have been considered in the simulations. The calculated stress profiles are presented in Figure 4. 

The solid curves in Figure 4 correspond to the results obtained with the elastic-plastic shots, while the dashed curves represent the ones of the rigid shots. In both cases, specific correlations between impact velocity, compressive residual stress, and peak depth occur: the increase in impact velocity leads to an increase in the maximum residual stress and its location (peak depth). This correlation has been identified and confirmed qualitatively in other studies, either experimental [30,31] or theoretical [32]. 

The results of the SP simulations with rigid shots are plotted with dashed curves in Figure 4. Though the residual stresses profiles are qualitatively similar to those of the elastic-plastic shot approach, the values alongside the peak depth seem absolutely unrealistic, confirming the initial assumption discussed in Section 2.4: that the rigid shot approximation may lead to significant errors. In particular, the calculated compressive residual stress peak values are 60% higher than the corresponding ones from the elastic-plastic approach. It should be mentioned that extraordinarily high impact stresses of approximately 1800 to 2000 MPa have been calculated at the top layers of the part when simulating SP processes with velocities of 77 and 65 m/s, respectively. Notice that these impact stresses exceed by far the ultimate tensile strength of the part material (1645 MPa) and would cause damage of these layers. Experimental investigations of SP processes with the same steel shots (S460) and part material (51CrV4) proved that the part material sustains these two velocities without damage. 

**Figure 5:**
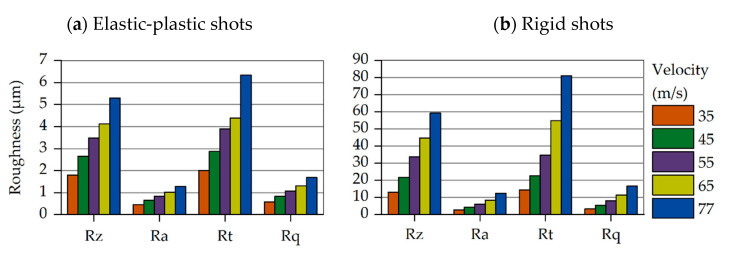
Roughness values for variable impact velocity: Elastic-plastic and rigid (non-deformable) shots approximation. (**a**) Elastic-plastic shots; (**b**) Rigid shots.

Figure 5 contains two bar charts summarizing the selected roughness parameters (Total Height, R_t_, Mean roughness, R_a_, Root Mean Square of roughness, R_q_, and Average Depth of roughness, R_z_). They have been determined for both approaches of the mechanical properties of the shots using Equations (6)–(8). 

The left chart in Figure 5 has been derived from the elastic-plastic shot approach and the right one from that with the rigid shots. The different colors stand for the various velocities. The increase in the surface roughness parameters with increasing impact velocity is easily recognizable. This is because the higher velocity yields a larger amount of energy transferred to the specimen, causing deeper imprints. Comparison of the results obtained from the two approaches reveals remarkable deviations between them. As expected, the imprint of the rigid shots to the part’s surface is significantly larger, yielding higher roughness parameters. At the highest velocities of 77 and 65 m/s, the rigid shot approach leads to approximately 10 times higher roughness parameter values compared to those resulting from the elastic-plastic shot approach.

### 3.2. Impact Angle

The random shot generation algorithm has been developed implementing the feature for an oblique shots stream. Therefore, an additional parametric study has been performed, using variable impact angle but a constant absolute shot velocity. Totally, six different angles were studied, covering a vertical impact (90 degrees) until an angle of 45 degrees. Therewith, the complete range of practical interest for serial automotive leaf spring applications has been covered. From the parametrical study shown in Section 3.1, it emerged that the impact velocity of 77 m/s causes a wider residual stresses profile with the largest peak value. Therefore, this velocity value was used, aiming to reveal most clearly the effect of the impact angle. The extracted results for both shot material approaches are presented in Figure 5.

**Figure 6:**
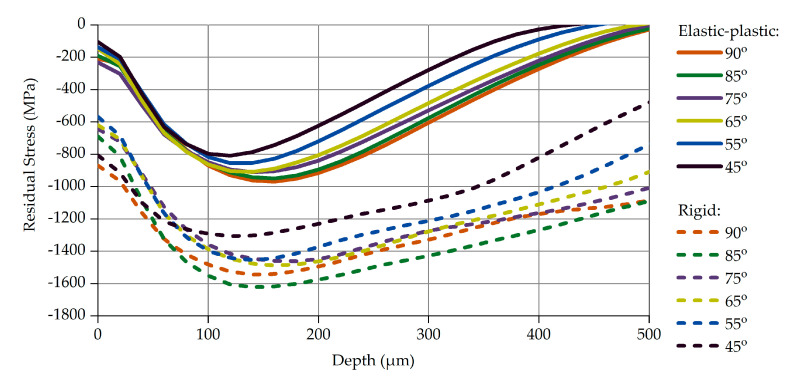
Compressive Residual Stress profiles, for various impact angles.

The curves for impact angles of 90 degrees shown in Figure 6 are identical to the ones for 77 m/s in Figure 5. Decreasing the impact angle leads to a decrease in both the maximum residual stress value and its peak depth. This observation confirms suggestions of theoretical SP process approximations, in which only the vertical component of the shot velocity is considered. Hence, an oblique shot stream can be equivalently reproduced with a vertical shot stream yielding almost identical results. 

As expected, and already discussed in Section 3.1, the simulations with rigid shots provide extraordinarily high residual stress values and quantitatively non-representative distributions at depths higher than the peak depth. On the other hand, the results of the elastic-plastic shot approach, especially the maximum stress value and peak depth, are much closer to experimental results from similar shot-part material combinations studied recently in [15].

**Figure 7:**
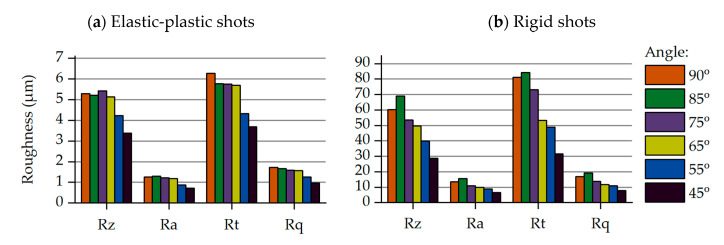
Roughness values for variable impact angle: elastic-plastic and rigid shots approximation. (**a**) Elastic-plastic shots; (**b**) Rigid shots.

Following the same logic as the previous parametric study in Section 3.1, the selected roughness values have been compared for each impact angle for both approaches. The calculated roughness parameters are summarized in the bar charts shown in Figure 7. Decreasing the impact angle, meaning that the vertical component of velocity is decreased too, lowers the deformations induced on the part’s surface. This effect is clearly visible at impact angles ranging from 45 to 65 degrees, while this effect becomes smoother or even vanishes at higher angles. The use of rigid shots leads to extraordinary roughness parameter values, since they are not capable of absorbing any dynamic energy during the impact. 

## 4. Validation and Discussion

The two-dimensional simulation of the SP process is certainly a simplification, and a validation of the results is necessary. Many studies resulted to semi-analytical models for induced residual stresses profiles by SP [32,33,34,35]. These studies follow the fundamentals of Hertzian elastic contact theory and Iliushin’s elastic-plastic theory, combined with concepts of hardening laws. They also adopt multiple approximations and simplifications, especially regarding the mechanical behavior of the materials, e.g., bilinear plasticity behavior of the peened specimens, or even a perfect plastic one.

Within the framework of the present work, the well-known equation of Ogawa and Asano [32], for the calculation of the maximum compressive residual stress, has been used to validate the results of the 2D simulations. Using the assumption of a bilinear plastic material, the abovementioned equation was formulated in [32]:

σ_rmax_^th^ = (0.333 + 0.286ab)(1 − ab)[(2ab − 1) σ_y_ − 1.038abρ^0.2^ E_eq_^0.8^ V^0.4^ ]. (9)

Herein, α is the ratio of Tangent to Young’s modulus; β is the ratio of plastic to elastic deformation, which can be either measured or approximated by calculations; σ_y_ and ρ are the flow stress and density of the shot material, respectively; E_eq_ is the equivalent elastic modulus for a Hertzian elastic contact; and V is the shot velocity. It is remarkable that this analytical equation does not consider the diameter of the shots. This is because Ogawa and Asano proved in the experimental part of their studies that the shot diameter does not significantly affect the maximum residual stress. 

**Figure 8:**
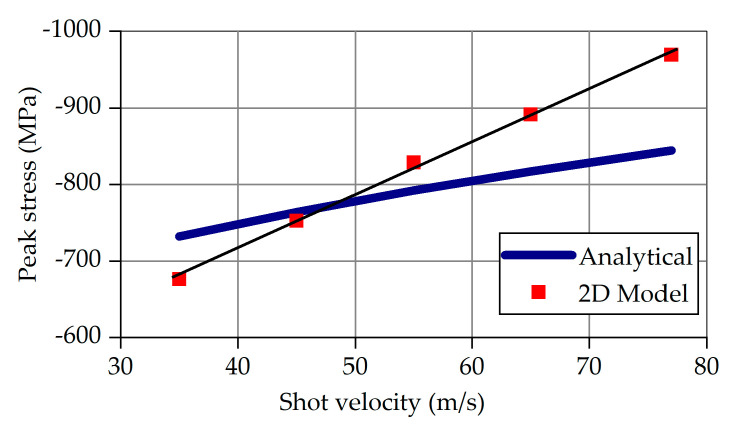
Analytical and numerical (2D model) values of the maximum compressive residual stress.

By simplifying the stress–strain curve of the target material into a bilinear one, the α ratio can be easily calculated. In addition, β which reflects the plastic over purely elastic contact radius, is assumed as 1. The other material parameters are known, both for the shots and the part. Therewith, the maximum compressive residual stresses at impact velocities V = 35–77 m/s can be calculated using Equation (5). The results are visualized and compared to the 2D model’s results in Figure 8.

The red marks in Figure 8 correspond to the 2D model’s results for the various velocities at vertical impact. Linear regression of the red marks yields the thin black line. The thick blue line represents the analytical results according to Equation (5). The deviations are acceptable, amounting from 9 to 13% for the lowest and the highest velocity, respectively.

**Figure 9:**
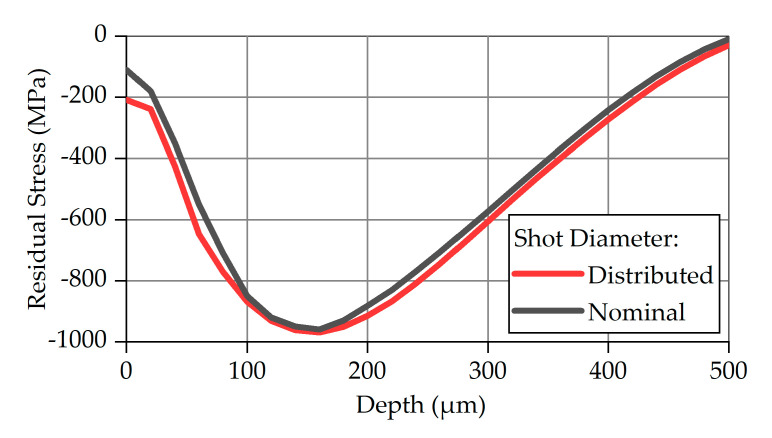
Calculated and simulated values for maximum compressive residual stress.

The necessity of modeling shots with a diameter distribution which matches one of the real shots has been investigated by FE simulations with shots having constant diameter equal to the nominal value of 1.4 mm. Notice that the number of the shots with nominal diameter differs from that with variable shot size. Equal in both cases is the total mass of the shots. In both simulations, the shots have a velocity of 77 m/s and an impact angle of 90°. The profiles of the calculated residual stresses are illustrated in Figure 9.

Since the material properties and the boundary conditions are identical, the peak value and the peak depth are almost the same—an observation which is in good agreement with the work of Ogawa and Asano [32]. The region between the specimen surface (0 mm) and the peak depth (~160 μm) exhibits the largest deviation in residual stresses: the residual stresses resulting from the variable shot diameters are larger by a factor from 10% to 20% at the same depth. This specific result can be attributed to the stress field which is developed when shots with a smaller diameter than the nominal one impact the specimen.

The comparison of the results shows the capability of the developed 2D model and confirms its satisfactory accuracy. Furthermore, the results of the parametrical studies are well in line with experimental ones from similar shot-part material combinations. Due to its reasonable background regarding the consideration of realistic shot material properties and shot size distributions, in conjunction with the qualitatively accurate simulation results within short calculation time, the model may be attractive to the industry to develop and/or optimize SP processes in a cost- and time-efficient manner. As an outlook, since not explicitly shown in this paper, the model can cover transient material properties and different hardening rules. Mechanical prestressing of the specimen (so-called Stress Shot Peening), which is nowadays common in the automotive spring industry, can be also easily implemented.

## 5. Summary and Conclusions

The 2D simulation has been proved to be a time-saving approach for parametrical studies on automotive leaf spring parts, since total simulation time here ranged around two and a half hours, using eight cores of an Intel Xeon © at 3.2 GHz. Elastic-plastic properties for both shots and part, full coverage, and a large peened area of the part (20 mm) have been considered. The developed 2D model allows the implementation of multiple shot diameter distributions. This is an important breakthrough on the scientific field, making it possible to consider mixtures of different shot types and/or distributions. This is the case in many industrial applications, e.g., in automotive leaf and coil springs and antiroll bars, which use new and used (deformed) shots in certain percentages. Such a case may lead to significant residual stress profile changes. 

The validation studies yielded qualitatively accurate residual stress profiles, with small errors in the maximum value. In addition, realistic roughness parameters have been extracted from the simulations. A rigid shot approach, which is often common in practice, should be always treated with caution. It is here assessed as unrealistic for shot-peening simulations on high-strength spring steels, where cast steel shots and part have comparable mechanical properties. The errors obtained in the present case study are large both qualitatively and quantitatively. Despite the existence of analytical-empirical equations, the developed 2D model is a valuable alternative that may be preferred, since it yields accurate results within reasonable computational time, implementing and accounting for all process parameters in a realistic way, e.g., variable shot diameters or even stochastic deviations in the material properties and variable surface properties. 

## Figures and Tables

**Table 1:** Sieve analysis of the S460 shots and corresponding data of the modeled shots.

Screen Size (mm)	Weight Retained—Nominal (%)	Weight Retained—Model (%)
2.00	0	0
1.70	<5	0
1.18	>85	93.06
1.00	>96	99.71

**Table 2:** FE model essential parameters.

Parameter	Value
Element type	Three and four node shells
Problem formulation	Plane strain
Number of elements (specimen)	~10^5^
Number of elements (shots)	~3 × 10^5^
Minimum element size	20 μm
Material type (specimen)	Multilinear elastoplastic
Material type (shots)	Bilinear elastoplastic
Friction coefficient	0 (not considered)
Boundary conditions (specimen)	Displacement restriction
Boundary conditions (shots)	Velocity

**Table 3:** Key material data for both specimen and shots.

Parameter	51CrV4	S460
Young modulus (GPa)	206	
Yield stress (MPa)	1450	1500
Ultimate Tensile Strength (MPa)	1645	1700

**Table 4:** Material composition of specimen and shots.

Material	C (%)	Mn (%)	Si (%)	P (%)	S (%)	Cr (%)	V (%)
**51CrV4**	0.47–0.55	0.7–0.11	max 0.4	max 0.025	max 0.025	0.9–1.2	0.1–0.25
**S460**	0.85–1.2	0.6–1.2	0.4–1.2	max 0.05	max 0.05	-	-

## Data Availability

Data sharing is not applicable to this article.

## Nomenclature

α	Mass proportional coefficient
β	Stiffness proportional coefficient
ε	Strain rate
ζ_i_	Damping coefficient for the i^th^ eigenmode
ρ	Material density
σ_rmax_^th^	Maximum compressive residual stress
σ_y_	Yield stress
ω_i_	Natural frequency for the i^th^ eigenmode
ω_0_	Minimal modal frequency
a	Ratio of Tangent to Young’s modulus
b	Ratio of plastic to elastic deformation
C	Damping matrix, Cowper-Symonds linear parameter
E	Young’s modulus
E_eq_	Equivalent Young’s modulus for a Hertzian elastic contact
E_t_	Tangent modulus
h	Height of the specimen
H	Plastic hardening modulus
K	Stiffness matrix
L	Roughness evaluation length
M	Mass matrix
q	Cowper–Symonds yield stress scale factor
R_a_	Root Mean Square of roughness
R_q_	Average Depth of roughness
R_t_	Mean Roughness
R_z_	Arithmetic average value of five total heights of R_t_
V	Shot velocity
Z(x)	Roughness profile height function along the *x*-axis
